# Associations Between Longitudinal Trajectories of Cognitive and Social Activities and Brain Health in Old Age

**DOI:** 10.1001/jamanetworkopen.2020.13793

**Published:** 2020-08-20

**Authors:** Melis Anatürk, Sana Suri, Enikő Zsoldos, Nicola Filippini, Abda Mahmood, Archana Singh-Manoux, Mika Kivimäki, Clare E. Mackay, Klaus P. Ebmeier, Claire E. Sexton

**Affiliations:** 1 Department of Psychiatry, University of Oxford, Warneford Hospital, Oxford, UK; 2 Wellcome Centre for Integrative Neuroimaging, Oxford Centre for Functional MRI of the Brain, Oxford, UK; 3 Nuffield Department of Clinical Neurosciences, University of Oxford, John Radcliffe Hospital, Oxford, UK; 4 Wellcome Centre for Integrative Neuroimaging, Oxford Centre for Human Brain Activity, University of Oxford, Warneford Hospital, Oxford, UK; 5 Department of Epidemiology and Public Health, University College London, London, UK; 6 Inserm U1153, Epidemiology of Ageing and Neurodegenerative Diseases, Université Paris-Descartes, Paris, France

## Abstract

**Importance:**

Prior neuroimaging studies have found that late-life participation in cognitive (eg, reading) and social (eg, visiting friends and family) leisure activities are associated with magnetic resonance imaging (MRI) markers of the aging brain, but little is known about the neural and cognitive correlates of changes in leisure activities during the life span.

**Objectives:**

To examine trajectories of cognitive and social activities from midlife to late life and evaluate whether these trajectories are associated with brain structure, functional connectivity, and cognition.

**Design, Setting, and Participants:**

This prospective cohort included participants enrolled in the Whitehall II study and its MRI substudy based in the UK. Participants provided information on their leisure activities at 5 times during calendar years 1997 to 1999, 2002 to 2004, 2006, 2007 to 2009, and 2011 to 2013 and underwent MRI and cognitive battery testing from January 1, 2012, to December 31, 2016. Data analysis was performed from October 7, 2017, to July 15, 2019.

**Main Outcome and Measures:**

Growth curve models and latent class growth analysis were used to identify longitudinal trajectories of cognitive and social activities. Multiple linear regression was used to evaluate associations between activity trajectories and gray matter, white matter microstructure, functional connectivity, and cognition.

**Results:**

A total of 574 individuals (468 [81.5%] men; mean [SD] age, 69.9 [4.9] years; median Montreal Cognitive Assessment score, 28 [interquartile range, 26–28]) were included in the present analysis. During a mean (SD) of 15 (4.2) years, cognitive and social activity levels increased during midlife before reaching a plateau in late life. Both baseline (global cognition: unstandardized β [SE], 0.955 [0.285], uncorrected *P* = .001; executive function: β [SE], 1.831 [0.499], uncorrected *P* < .001; memory: β [SE], 1.394 [0.550], uncorrected *P* = .01; processing speed: β [SE], 1.514 [0.528], uncorrected *P* = .004) and change (global cognition: β [SE], −1.382 [0.492], uncorrected *P* = .005, executive function: β [SE], −2.219 [0.865], uncorrected *P* = .01; memory: β [SE], −2.355 [0.948], uncorrected *P* = .01) in cognitive activities were associated with multiple domains of cognition as well as global gray matter volume (β [SE], −0.910 [0.388], uncorrected *P* = .02). Baseline (β [SE], 1.695 [0.525], uncorrected *P* = .001) and change (β [SE], 2.542 [1.026], uncorrected *P* = .01) in social activities were associated only with executive function, in addition to voxelwise measures of functional connectivity that involved sensorimotor (quadratic change in social activities: number of voxels, 306; *P* = 0.01) and temporoparietal (linear change in social activities: number of voxels, 16; *P* = .02) networks. Otherwise, no voxelwise associations were found with gray matter, white matter, or resting-state functional connectivity. False discovery rate corrections for multiple comparisons suggested that the association between cognitive activity levels and executive function was robust (β [SE], 1.831 [0.499], false discovery rate *P* < .001).

**Conclusions and Relevance:**

The findings suggest that a life course approach may delineate the association between leisure activities and cognitive and brain health and that interventions aimed at improving and maintaining cognitive engagement may be valuable for the cognitive health of community-dwelling older adults.

## Key Points

**Question:**

Are longitudinal trajectories of cognitive and social activities associated with brain structure and function?

**Findings:**

In this cohort study of 574 adults followed up for a mean of 15 years, the level of cognitive activities was associated with measures of cognitive function but not magnetic resonance imaging measures of brain structure or functional connectivity after corrections for multiple comparisons.

**Meaning:**

The findings suggest that a life course approach may better delineate the association between leisure activities and cognitive and brain health, with this study identifying cognitive activities as potential targets for intervention studies.

## Teaser

This cohort study examines trajectories of cognitive and social activities from midlife to late life and evaluates whether these trajectories are associated with brain structure, functional connectivity, and cognition.

## Introduction

Cognitively and socially enriched lifestyle may be associated with preservation of brain health at older ages.^1,2^ For example, greater participation in cognitive activities is associated with higher cognitive performance and a lower risk of dementia.^3^ Furthermore, social isolation is a potential risk factor for cognitive impairment^4^ and Alzheimer disease.^5^ Previous work^6^ that used magnetic resonance imaging (MRI) suggests that intellectually and socially engaging activities are associated with greater global white matter (WM) volume, fewer WM lesions, and larger hippocampi. Functional MRI has shown that activity levels correlate with brain function,^7,8^ including reduced blood oxygenation level–dependent activation in the frontal cortex.^8^ Although MRI studies offer valuable insights into the biological characteristics of the associations of activity with cognition, the present evidence is often limited to single assessments of activities. Consequently, leisure activities are largely treated as static constructs over time.^9^

Although longitudinal modeling techniques (eg, growth curve models) may be better suited to examine whether activity patterns during the life span are associated with brain structure and function, these methods are underused in MRI studies.^9^ Because leisure activities change over time,^10,11^ with interindividual differences in the level and change in activity levels,^12,13,14^ we aimed to evaluate whether individuals may be divided into subgroups based on repeat measures of cognitive and social activity levels and whether activity trajectories are associated with MRI measures of brain structure and functional connectivity in late life. On the basis of previous findings,^12,13,14^ 5 trajectory subgroups were hypothesized. We also hypothesized that higher participation in cognitive and social activities over time would be associated with greater gray matter (GM) and WM volume, better WM integrity, and fewer WM lesions.^6^ No a priori hypotheses were developed with regard to resting-state functional connectivity because of limited evidence available. An exploratory analysis was therefore performed of networks implicated in aging and cognitive decline,^15,16^ namely, the default mode network, executive control network, and frontoparietal network. On the basis of prior work,^3,17^ higher cognitive and social engagement over time was hypothesized to be associated with greater levels of global cognition, executive function, memory, and processing speed.

## Methods

### Design and Setting

This cohort study used data from the Whitehall II study,^18^ which began in 1985 and is a prospective occupational cohort study that investigates social gradients in health outcomes. In brief, a total of 10 308 British civil servants 35 to 55 years of age were recruited at baseline (phase 1, 1985-1988). More recently, 800 participants (12.7%) were randomly selected from phase 2 (2011-2013) to participate in the Whitehall II imaging substudy at the Centre for Functional Magnetic Resonance Imaging of the Brain (FMRIB), University of Oxford.^19^ The current analyses used leisure activity information collected in calendar years 1997 to 1999, 2002 to 2004, 2006, 2007 to 2009, and 2011 to 2013 and MRI and cognitive data from January 1, 2012, to December 31, 2016. For an overview of the included study phases, see eFigure 1 in the Supplement. This study received ethical approval from the University of Oxford Central University Research Ethics Committee and the University College London Medical School Committee on the Ethics of Human Research. All participants gave written informed consent. All data were deidentified. This study followed the Strengthening the Reporting of Observational Studies in Epidemiology (STROBE) reporting guideline.^20^

### Study Sample

The sample consisted of 574 individuals (the Table provides sample characteristics) who had completed the activity questionnaire in at least 4 of the 5 data waves and had no more than 1 item missing from each completed activity questionnaire. For a flowchart of participant selection and exclusion, see eFigure 2 in the Supplement.

**Table.** Sample Characteristics

Characteristic	Finding (N = 574)^a^
Demographic	
Age at MRI, y	69.6 (4.9) [60.6-84.4]
Sex, No. (%)	
Male	468 (81.5)
Female	106 (18.5)
Educational level^b^	3.54 (1.06) [1-5]
Activities	
Cognitive activity at baseline	1.02 (0.35) [0-2.35]
Social activity at baseline	1.04 (0.44) [0-2.36]
Global cognition	
MoCA score, median (IQR) [range]	28 (26-28) [18-30]
MoCA score <26	103 (17.9)
MoCA score ≥26	471 (82.1)
Executive function	
Digit span: forward	11.12 (2.28) [5-16]
Digit span: backward	9.77 (2.44) [4-16]
Digit span: sequence	10.15 (2.42) [1-16]
Category fluency	22.66 (5.39) [7-40]
Letter fluency	15.88 (4.45) [3-31]
TMT B, median (IQR) [range], s^c^	57 (44-74) [24-289]
Memory	
HVLT-R	
Total recall	27.88 (4.41) [11-36]
Delayed recall, median (IQR) [range]	10 (8-10) [0-12]
RDI, median (IQR) [range]	11 (10-11) [5-12]
RCF	
Immediate recall	16.26 (6.36) [0-33]
Delayed recall	15.92 (6.01) [0-31]
Recognition	8.60 (1.87) [1-12]
Processing speed	
Digit coding score	63.8 (12.90) [24-114]
RT, median (IQR) [range], ms^c^	
Simple	299.47 (272.98-334.90) [216.76-837.31]
Choice	331.09 (303.62-366.74) [244.33-586.20]
MT, median (IQR) [range], ms^c^	
Simple	256.79 (217.75-308.62) [139.93-681.13]
Choice	276.74 (238.53-327.03) [122.79-637.12]
TMT A, median (IQR) [range], s^c^	28 (23-34) [13-92]
Structural MRI measures	
Brain volumes, % of ICV	
Global GM volume	38.37 (1.94) [29.20-44.49]
Global WM volume	37.85 (2.33) [31.82-44.09]
CSF volume	23.79 (3.11) [16.45-35.02]
Global WM hyperintensities, median (IQR) [range]	0.4 (0.29-0.51) [0.08-2.17]
FA	0.48 (0.02) [0.41-0.54]
AD, ×10^3^/mm^2^/s	1.08 (0.02) [1.01-1.19]
MD, ×10^3^/mm^2^/s	0.68 (0.03) [0.61-0.80]
RD, ×10^3^/mm^2^/s	0.49 (0.03) [0.40-0.61]
Functional MRI measures	
Relative motion, mm	0.17 (0.09) [0.01-0.60]
Intranetwork functional connectivity	
Anterior DMN	18.77 (5.82) [5.99-44.58]
Precuneus DMN	16.75 (5.32) [4.77-36.70]
Posterior DMN	13.28 (3.49) [3.56-24.38]
ECN, median (IQR) [range]	11.77 (9.41-14.49) [4.45-35.08]
Left FPN	17.84 (5.02) [7.19-35.17]
Right FPN	16.81 (4.33) [6.19-35.93]

Abbreviations: AD, axial diffusivity; CSF, cerebrospinal fluid; DMN, default mode network; ECN, executive control network; FA, fractional anisotropy; FPN, frontoparietal network; GM, gray matter; HVLT-R, Hopkins Verbal Learning Test Revised; ICV, intracranial volume; IQR, interquartile range; MD, mean diffusivity; MoCA, Montreal Cognitive Assessment; MRI, magnetic resonance imaging; MT, movement time; RCF, Rey-Osterrieth Complex Figure; RD, radial diffusivity; RDI, recognition discrimination index; RT, reaction time; TMT A, Trail Making Test Part A; TMT B, Trail Making Test Part B; WM, white matter.

^a^ Data are presented as mean (SD) [range] unless otherwise indicated.

^b^ For education, the scale was as follows: 1, no qualifications; 2, O-levels or equivalent (at 16 years); 3, A-levels, college certificate, or professional qualification (at 18 years or older); 4, bachelor degree; 5, higher degree.

^c^ Raw values are reported, although scores were reverse coded for the analyses.

### Assessment of Leisure Activities

Respondents indicated how frequently they had participated in 13 activities (eTable 1 in the Supplement) during the past 12 months on a 4-point scale (0 indicating never and 3 indicating weekly). Weights were assigned to each activity based on their relative cognitive or social demand. A weighted mean score was computed (range, 0-3) to reflect social and cognitive activity engagement, in which higher values indicated greater levels of engagement.

### Cognitive Function

A cognitive battery was administered face to face before the MRI. In accordance with previous studies,^21,22^ individual test scores were *z* transformed and summed to form 3 key subdomains: executive function, memory, and processing speed. Global cognition was also assessed using the Montreal Cognitive Assessment (MoCA).^23^ A description of each test used is available in the eMethods and eTable 2 in the Supplement.

### MRI Data Acquisition

The MRI data were collected using a 3-T Siemens Magnetom Verio (April 1, 2012, to December 31, 2015) or a 3-T Siemens Magnetom Prisma (June 1, 2015, to December 31, 2016), with a 32-channel head coil. Scanning was undertaken at the FMRIB at the University of Oxford. In brief, T1-weighted (GM volume), diffusion-weighted (WM microstructure), resting-state functional, and fluid-attenuated inversion recovery (WM lesions) images were used. Details on the MRI acquisition parameters are given in the eMethods in the Supplement.^19^

### MRI Data Preprocessing Steps

Structural and functional images were preprocessed using tools from the FMRIB Software Library.^24^ The eMethods in the Supplement provide additional information on the preprocessing steps of MRI data.

### Statistical Analysis

Data were analyzed from October 7, 2017, to July 15, 2019. To identify longitudinal trajectories from repeat measures of activity levels, latent growth curve models and latent class growth models were performed (MPlus, version 8 [Muthén & Muthén]). Latent growth curve models were used to estimate a single mean trajectory across the sample while also estimating individual variability around this mean trajectory.^25^ Latent class growth models were applied to evaluate whether participants could be classified into multiple trajectory groups.^26^ We also assessed different patterns of change in activities, including no change, linear change, and quadratic change over time. Models were then compared to evaluate the best fit (further details are given in the eMethods in the Supplement). Restricted maximum likelihood with robust SEs was also used because this estimator is robust to deviations from normality.^27^ Full information maximum likelihood was used to estimate parameters in the presence of missing data.

To evaluate whether longitudinal trajectories of leisure activities are associated with measures of brain structure, functional connectivity, and cognition, general linear models were used. The dependent variables included voxelwise and global measures of GM volume (FMRIB Software Library voxel-based morphometry),^28^ WM microstructure (Tract-Based Spatial Statistics),^29^ and functional connectivity (dual regression).^30^ For an overview of the resting-state networks, see eFigure 3 in the Supplement. All voxelwise statistics were performed with the FSL randomize^31^ tool (5000 permutations) with Threshold-Free Cluster Enhancement^32^ and familywise error–corrected *P* values (for multiple comparisons across space). Analyses of imaging outcomes were adjusted for age (at the time of scan), sex, educational level, scanner model, and relative head motion. These covariates (excluding scanner and motion) were also included in the analyses of cognitive outcomes. Linear regression with cognitive outcomes or derived MRI outcomes (eg, total GM) were examined in SPSS software, version 25 (SPSS Inc). All tests were 2-sided, with uncorrected *P* values (ie, uncorrected *P* < .05) and false discovery rate (FDR)–corrected *P* values reported. Syntax for all analyses (apart from voxelwise) are available in eTables 3-5 in the Supplement.

Because several resting-state networks are known to reflect age sensitivity,^33,34^ we evaluated whether networks (outside those of interest) were associated with activity levels, including sensorimotor, visual, and temporoparietal networks. To examine whether significant associations were moderated by cognitive status (healthy vs impaired), interaction terms were entered into the linear regression (described in the eMethods in the Supplement) in a series of follow-up analyses.

## Results

### Sample Characteristics

The descriptive statistics for included participants are reported in the Table. A total of 574 participants were included in the main analyses, with a mean (SD) age of 69.6 (4.9) years (age range, 60.6-84.4 years) at the MRI examination (2012-2016). Of the total sample, 468 (81.5%) were men. Cognitive and social activity levels were on measured repeatedly over a mean (SD) period of 15 years (4.2). Overall, the median MoCA score was 28 (interquartile range, 26-28), with 103 individuals (17.9%) scoring below 26, which is an established cutoff for cognitive impairment. For a comparison of included to excluded participants, see eResults and eTable 6 in the Supplement.

### Leisure Activity Trajectories

**Figure 1.**
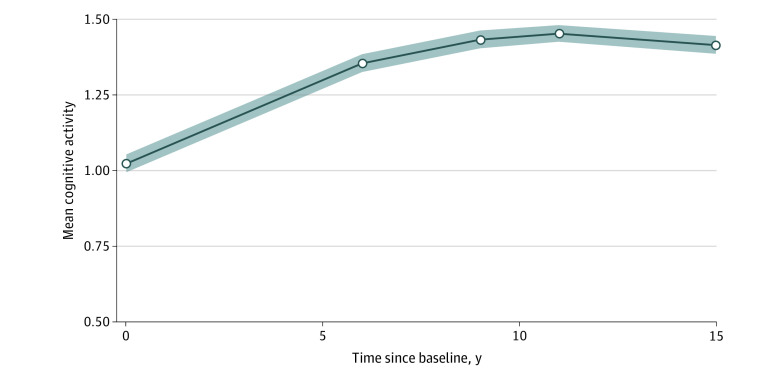
Means of Cognitive Activity Levels Over Time, as Estimated by the Quadratic Growth Curve Model Data are presented as intercept coefficients. Shaded areas indicate 95% CIs.

**Figure 2.**
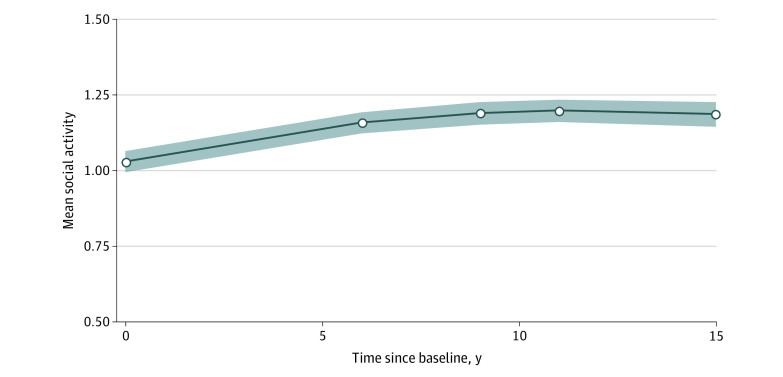
Means of Social Activity Levels Over Time, as Estimated by the Quadratic Growth Curve Model Data are presented as intercept coefficients. Shaded areas indicate 95% CIs.

The quadratic latent growth curve model provided the most adequate fit for cognitive activity levels (comparative fit index, 0.967; Tucker-Lewis index, 0.967; root mean square error of approximation, 0.081 [95% CI, 0.058-0.104]) (eTables 7 and 8 in the Supplement). The quadratic latent growth curve model also provided adequate fit for social activity levels measured over time (comparative fit index, 0.909; Tucker-Lewis index, 0.909; root mean square error of approximation, 0.145 [95% CI, 0.123-0.167]). Figure 1 and Figure 2 show the estimated activity trajectories.

### Cognitive Function

**Figure 3.**
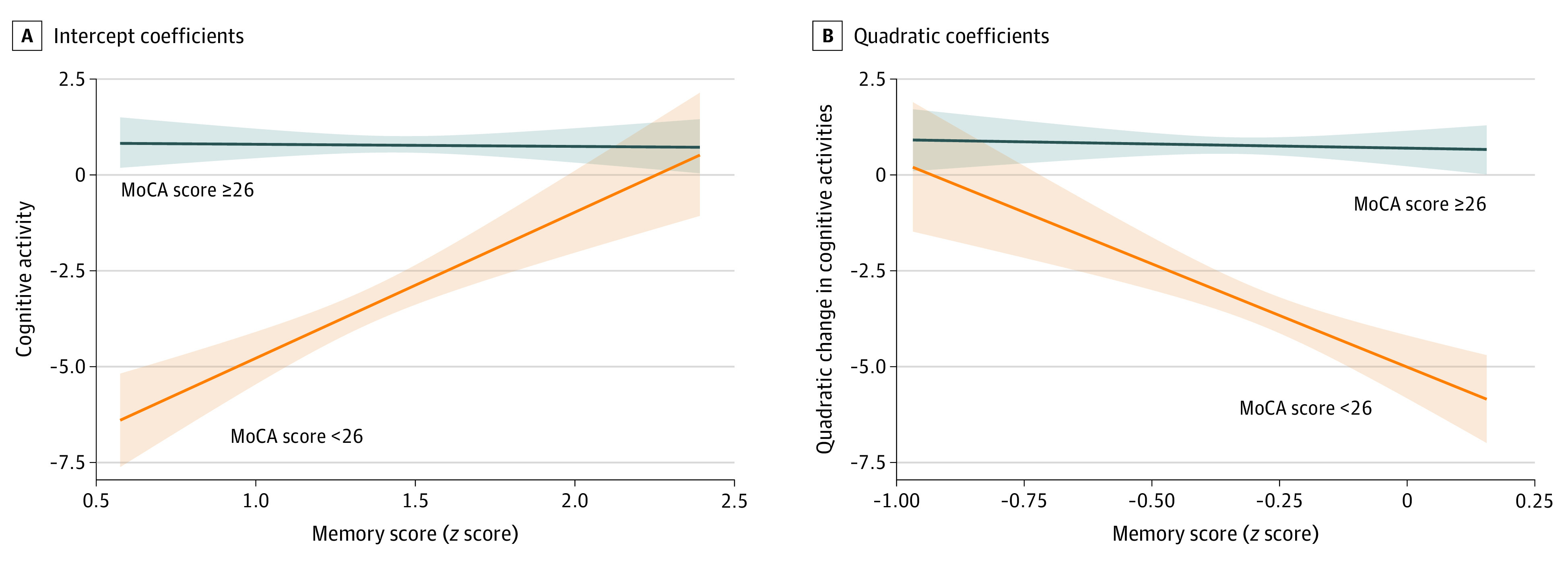
Interaction Between Montreal Cognitive Assessment (MoCA) Scores and Cognitive Activities for Memory Scores Data are presented as intercept coefficients (A) and quadratic coefficients (B). Unadjusted associations are presented, with shaded areas reflecting 95% CIs.

Estimates of each individual’s trajectories (ie, intercepts and linear and quadratic coefficients) were used as regressors in linear regressions of cognitive and MRI markers (complete results are given in eTables 10 and 11 in the Supplement). Intercepts of cognitive activities were significantly and positively associated with global cognition (β [SE], 0.955 [0.285], uncorrected *P* = .001), executive function (β [SE], 1.831 [0.499], uncorrected *P* < .001), memory (β [SE], 1.394 [0.550], uncorrected *P* = .01), and processing speed (β [SE], 1.514 [0.528], uncorrected *P* = .004). The quadratic coefficients for cognitive activities were negatively associated with global cognition (β [SE], −1.382 [0.492], uncorrected *P* = .005), executive function (β [SE], −2.219 [0.865], uncorrected *P* = .01), and memory (β [SE], −2.355 [0.948], uncorrected *P* = .01). Although higher cognitive engagement was associated with greater cognitive performance in late life, trajectories of an initially steeper increase and a subsequent leveling or decline in cognitive engagement were associated with better performance on global and domain-specific cognitive function. A significant interaction (eTable 11 in the Supplement) was found between cognitive status and intercepts (β [SE], 3.828 [1.259], uncorrected *P* = .002) and quadratic (β [SE], −5.179 [2.141], uncorrected *P* = .02) coefficients of cognitive activities. Plots of the interaction suggested that the associations between cognitive activity intercepts (Figure 3A) and quadratic coefficients (Figure 3B) were only observed for individuals with cognitive impairment.

For social activities, intercepts (B [SE], 1.695 [0.525], uncorrected *P* = .001) and quadratic coefficients (B [SE], 2.542 [1.026], uncorrected *P* = .01) were positively associated with executive function. Therefore, increases in social activity levels were associated with better executive function in late life, although trajectories characterized by an initial steeper incline and subsequent steeper decline in cognitive engagement were associated with poorer cognitive function. No other associations, including post hoc interactions, were significant.

### Brain Structure

Quadratic coefficients of cognitive activities were negatively associated with total GM volume (B [SE], −0.910 [0.388], uncorrected *P* = .02). Trajectories of a steeper initial increase and subsequent leveling or decline in cognitive activities were associated with higher total GM volume at follow-up. Neither activity type demonstrated any associations with global or voxelwise indexes of WM microstructure or with global WM lesions or interaction with MoCA group (eTables 9 and 10 in the Supplement).

### Functional Connectivity

**Figure 4.**
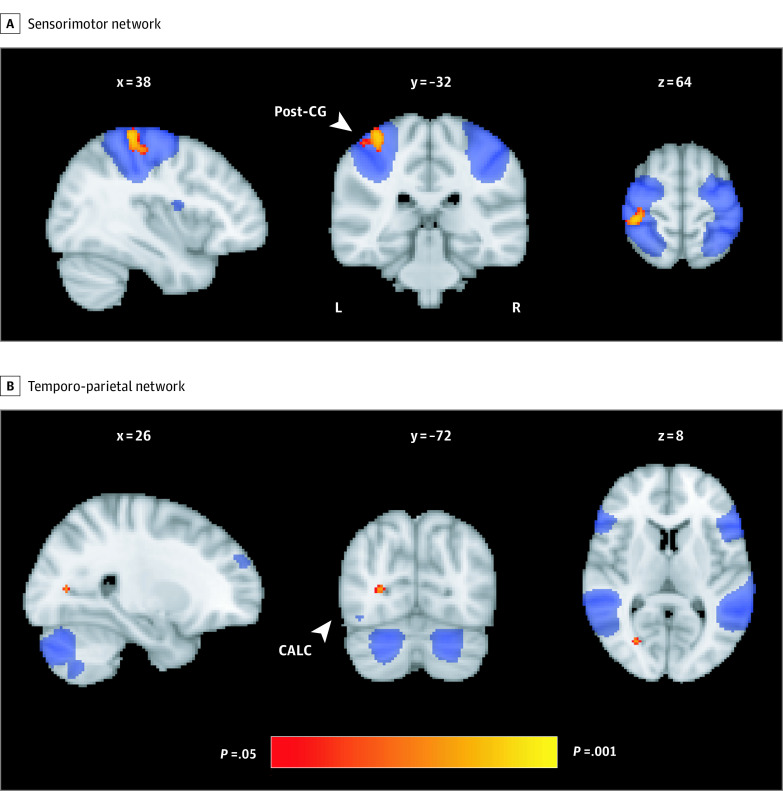
Results of Voxelwise Regressions Between Social Activity Trajectories and Resting-State Functional Connectivity A, Negative correlation between quadratic coefficients of social activities and functional connectivity of the sensorimotor network. B, Negative correlation between linear coefficients of social activities and functional connectivity of the temporo-parietal network. These clusters were significant after covarying for age, sex, education, scanner, and head motion and are overlaid on spatial maps of the sensorimotor network (A) and temporo-parietal network (B) and MNI152 template. The x, y, and z coordinates are reported in MNI space (millimeters); these results did not survive false discovery rate corrections. CALC indicates intracalcarine cortex; post-CG, postcentral gyrus.

No associations or interactions with MoCA status were identified between cognitive or social activities and global or voxelwise indexes of frontoparietal network, default mode network, and executive control network functional connectivity (eTables 9 and 10 in the Supplement). However, post hoc analyses of other age-sensitive resting-state networks found a negative association between quadratic coefficients of social activities and functional connectivity of the sensorimotor network and the right postcentral gyrus (number of voxels = 306; uncorrected *P* = .01) (eTable 12 in the Supplement). A significant inverse association was found between linear coefficients of social activities and functional connectivity between the temporoparietal network and the intracalcarine cortex (number of voxels = 16; uncorrected *P* = .02) (Figure 4B).

### FDR-Corrected Results

FDR corrections were applied across the main analyses. The positive association between cognitive activity intercepts and executive function (β [SE], 1.831 [0.499], FDR-corrected *P* < .001) was the only result to remain significant.

## Discussion

We report findings from, to our knowledge, the first multimodal MRI study to examine whether cognitive and social activity trajectories are associated with brain integrity in late life. Overall, we found that activity levels slightly increased during midlife before subsequent leveling in early late life. We found positive associations between activity trajectories and cognitive function. Cognitive status also appeared to moderate the associations between activity trajectories and memory performance. Quadratic coefficients of cognitive activities were negatively associated with global GM volume and functional connectivity of 2 resting-state networks. After FDR corrections, the only association detected was between cognitive activity intercepts and executive function.

This study found no evidence of trajectory subgroups based on self-reported activity levels. Instead, a single-group solution provided the best fit, suggesting that all Whitehall II study participants were relatively homogenous in their social and cognitive engagement levels during the study period. These results are discrepant from prior studies that also used mixture modeling and converged on a 5-class linear solution for social engagement^13,14^ and leisure activity trajectories.^35^ Key differences in sample characteristics (eg, age and follow-up length) and study design (eg, type of model applied) and method used (latent class growth modeling vs growth mixture modeling) may account for the differences between the present findings and those of the prior studies. Our results further suggest that activity levels slightly increased during midlife, which was followed by a subsequent decrease in early late life. These observed increases in activity levels may be associated with the onset of retirement and uptake of new activities and/or more frequent participation in activities enjoyed earlier in life.^36^

Prior evidence indicates an association between cognitive activities and late-life cognitive function in older adults.^37^ The results from the present study were consistent with these observations, suggesting that cognitive activity levels (ie, intercepts) are positively associated with multiple domains of cognition. The positive association between cognitive activity levels and memory seemed to be strongest among individuals with cognitive impairment, indicating that individuals with cognitive impairment may benefit from interventions designed to improve cognitive engagement. Increases in cognitive activities over time have previously been associated with better performance on tests of semantic knowledge, memory, language fluency, and reasoning.^11^ The results of the present study suggest a more complex association because increases in cognitive activities during midlife that were maintained or reduced during subsequent years were associated with higher performance on tests of global cognition and subdomains (eg, executive function, memory, and processing speed). Similarly, these trajectories were also associated with greater total GM volume. Although speculative, these results suggest that if intellectual engagement levels were improved during midlife, the potential benefits to cognitive and brain integrity would be maintained, even if these levels are not fully sustained over time. However, these findings should be interpreted with caution. These associations have not been reported previously, and in this study, only the association between cognitive activity levels and executive function was maintained after FDR corrections for multiple testing. Future follow-up studies will therefore be crucial for confirming whether the associations reported at an uncorrected *P* value are replicable. If replicated, the next step will be to evaluate how changes in different types of activities directly influence cognitive function over time.

Social activity levels have previously been shown to be associated with cognition, including global cognition, executive function, and memory.^17^ Increases in social activity also appear to be associated with less decline in semantic knowledge and better language fluency, reasoning, and memory over time.^38^ This study, however, suggests a more limited role of social engagement in maintaining cognition. Although higher levels of social activity were positively associated with executive function, trajectories of a steep initial increase and then a subsequent leveling or decrease in social activities were associated with poorer integrity of this subdomain at follow-up. Our results suggest that although increases in social activities from an individual’s baseline may contribute to better cognitive function in late life, taking on more activities than can be maintained over time could potentially be associated with negative consequences for executive function. Given that, to our knowledge, no prior study has found this pattern of results, it is important that future studies also examine how nonlinear changes in specific activities are associated with cognitive function over time. Provided that our results are replicated, it may be sensible to recommend that any lifestyle-based interventions developed to promote healthy aging are designed to be age adaptable.

Trajectories of social activities with a more curved pattern were associated only with higher functional connectivity between the sensorimotor network and right postcentral gyrus, in addition to between the calcarine cortex and temporoparietal networks. These findings are difficult to interpret, although may serve to highlight 2 networks of interest to future studies examining the effects of social activities on resting-state functional connectivity. We did not find any other associations between activities and the aging brain. These results are consistent with previous neuroimaging studies that reported null findings between midlife or lifetime activities and late-life GM volume,^39,40^ WM integrity,^39,40^ and WM lesions.^41,42^ The absence of significant findings may be attributable to other neural mechanisms, such as the buildup of β-amyloid plaques and hypometabolism.^42,43,44^ Several studies disagree with the present results and instead suggest that the associations between activities and the brain are widespread, spanning across multiple modalities.^7,41,45,46^ Longitudinal changes in brain structure and functional connectivity are potentially more sensitive correlates of social and cognitive engagement.^45^ Future longitudinal MRI studies are therefore required to evaluate whether the present results are replicable. Given that some individuals appeared to have improved activity levels over time,^10^ future comparisons in the MRI and cognitive profiles of these individuals with those who have declines in activities may be warranted.

### Strengths and Limitations

This study has strengths. The study included a well-characterized and large sample of adults, used multimodal imaging, and repeated assessment of 5 different activities, over a mean span of 15 years. These factors allowed a more detailed examination of the association between leisure activities and brain structure, functional connectivity, and cognition than has been possible in previous publications.

This study also has limitations. The study used a study-specific self-report questionnaire. A healthy volunteer effect was also observed,^47^ with those who remained in the study being significantly younger, having higher MoCA scores, and being more highly educated compared with excluded individuals (eTable 6 in the Supplement). A healthier-than-average sample may have undermined the ability to detect the hypothesized associations by reducing the variability present in the sample. Another consideration is that because of multicollinearity, mutual adjustment for intercept and quadratic coefficients of cognitive activities was not possible in the main analyses. This limitation may make the reported associations more difficult to interpret; for example, the association between cognitive activity levels and cognition may be attributable to the quadratic change in trajectories rather than the level at a given time point. In addition, no causality can be inferred based on the activity-cognition findings reported because of the observational nature of the study.

## Conclusions

This study found associations between cognitive activities and executive function. The study adds to an increasing body of evidence to support the funding of community-based programs that promote cognitive engagement among older adults to promote lifelong cognitive well-being.

## Notes

Supplement. **eMethods.** Supplementary Methods **eResults.** Supplementary Results **eFigure 1.** A Timeline of the Study Phases and Variables Included in the Present Analyses **eFigure 2.** Flowchart of Participant Selection and Exclusion **eFigure 3.** ICA Components Representing the Executive Control, Default Model and Fronto-Parietal Networks **eTable 1.** Social and Cognitive Demand Ratings for Each of the Items of the WHII Activity Questionnaire **eTable 2.** A Description of Each Test Within the Whitehall II Cognitive Battery **eTable 3.** MPLUS Syntax Used to Generate a Quadratic Latent Growth Curve Model (LGCM) **eTable 4.** MPLUS Syntax Used to Produce a 3-Class Linear Latent Class Growth Model (LCGM) **eTable 5.** SPSS Syntax Used for a Linear Regression With the Intercept, Linear and Quadratic Coefficients of Social Activity as the Predictors of Interest and FA as the Outcome Variable **eTable 6.** Comparisons of Included and Excluded Participants **eTable 7.** Model Fit Indices for All of the Unconditional Latent Growth Curve Models Assessed **eTable 8.** As the Quadratic Latent Growth Curve Model Best Described the Pattern of Growth in the Sample, the Table Demonstrates the Fit Indices for All of the Unconditional Quadratic Latent Class Growth Models **eTable 9.** Regression Coefficients for Cognitive Activities **eTable 10.** Regression Coefficients for Social Activities **eTable 11.** Interactions Between MoCA Group (MoCA Score <26 = 471; MoCA Score ≥26 = 103) And Activity Trajectories **eTable 12.** Social Activity Trajectories Were Negatively Correlated With Voxel-Wise Measures of Functional Connectivity Involving the Sensorimotor and Temporo-Parietal Networks **eReferences**
