# Supplementary material for: Associations Between Longitudinal Trajectories of Cognitive and Social Activities and Brain Health in Old Age
Source: JAMA Netw Open. 2020 Aug 20;3(8):e2013793. doi: 10.1001/jamanetworkopen.2020.13793 (PMC7441365; doi:10.1001/jamanetworkopen.2020.13793)
Supplement: Supplement. — eMethods. Supplementary Methods eResults. Supplementary Results eFigure 1. A Timeline of the Study Phases and Variables Included in the Present Analyses eFigure 2. Flowchart of Participant Selection and Exclusion eFigure 3. ICA Components Representing the Executive Control, Default Model and Fronto-Parietal Networks eTable 1. Social and Cognitive Demand Ratings for Each of the Items of the WHII Activity Questionnaire eTable 2. A Description of Each Test Within the Whitehall II Cognitive Battery eTable 3. MPLUS Syntax Used to Generate a Quadratic Latent Growth Curve Model (LGCM) eTable 4. MPLUS Syntax Used to Produce a 3-Class Linear Latent Class Growth Model (LCGM) eTable 5. SPSS Syntax Used for a Linear Regression With the Intercept, Linear and Quadratic Coefficients of Social Activity as the Predictors of Interest and FA as the Outcome Variable eTable 6. Comparisons of Included and Excluded Participants eTable 7. Model Fit Indices for All of the Unconditional Latent Growth Curve Models Assessed eTable 8. As the Quadratic Latent Growth Curve Model Best Described the Pattern of Growth in the Sample, the Table Demonstrates the Fit Indices for All of the Unconditional Quadratic Latent Class Growth Models eTable 9. Regression Coefficients for Cognitive Activities eTable 10. Regression Coefficients for Social Activities eTable 11. Interactions Between MoCA Group (MoCA Score <26 = 471; MoCA Score ≥26 = 103) And Activity Trajectories eTable 12. Social Activity Trajectories Were Negatively Correlated With Voxel-Wise Measures of Functional Connectivity Involving the Sensorimotor and Temporo-Parietal Networks eReferences [file jamanetwopen-3-e2013793-s001.pdf]

Supplementary Online Content

Anatürk M, Suri S, Zsoldos E, et al. Associations between longitudinal trajectories of cognitive and social activities and brain health in old age. *JAMA Netw Open*. 2020;3(8):e2013793. doi:10.1001/jamanetworkopen.2020.13793

eMethods. Supplementary Methods
eResults. Supplementary Results
eFigure 1. A Timeline of the Study Phases and Variables Included in the Present Analyses
eFigure 2. Flowchart of Participant Selection and Exclusion
eFigure 3. ICA Components Representing the Executive Control, Default Model and Fronto-Parietal Networks
eTable 1. Social and Cognitive Demand Ratings for Each of the Items of the WHII Activity Questionnaire
eTable 2. A Description of Each Test Within the Whitehall II Cognitive Battery
eTable 3. MPLUS Syntax Used to Generate a Quadratic Latent Growth Curve Model (LGCM)
eTable 4. MPLUS Syntax Used to Produce a 3-Class Linear Latent Class Growth Model (LCGM)
eTable 5. SPSS Syntax Used for a Linear Regression With the Intercept, Linear and Quadratic Coefficients of Social Activity as the Predictors of Interest and FA as the Outcome Variable
eTable 6. Comparisons of Included and Excluded Participants
eTable 7. Model Fit Indices for All of the Unconditional Latent Growth Curve Models Assessed
eTable 8. As the Quadratic Latent Growth Curve Model Best Described the Pattern of Growth in the Sample, the Table Demonstrates the Fit Indices for All of the Unconditional Quadratic Latent Class Growth Models
eTable 9. Regression Coefficients for Cognitive Activities
eTable 10. Regression Coefficients for Social Activities
eTable 11. Interactions Between MoCA Group (MoCA Score <26 = 471; MoCA Score ≥26 = 103) And Activity Trajectories
eTable 12. Social Activity Trajectories Were Negatively Correlated With Voxel-Wise Measures of Functional Connectivity Involving the Sensorimotor and Temporo-Parietal Networks
eReferences

This supplementary material has been provided by the authors to give readers additional information about their work.

© 2020 Anatürk M et al. *JAMA Network Open.*

eMethods. Supplementary Methods

Cognitive and social activity: As activities vary in their cognitive and social requirements,[1] we weighted each item by their relative demand. Item weightings were independently assigned by three authors (MA, CES, KPE), with disagreements resolved through discussion (for agreed ratings, see e*Table 1*). The weights assigned consisted of 'low' (= 0), 'medium' (= 1) or 'high' (= 2) cognitive/social demand. Therefore, an item with a higher social/cognitive demand weighting provided a greater contribution towards the overall social/cognitive engagement score, compared to an activity rated as less demanding. A weighted mean[2] was then calculated as follows:

$$\frac{(item_1 * weight) + (item_2 * weight_2) \ldots (item_n * weight_n)}{weight_1 + weight_2 + \ldots weight_n}$$

This produced summary scores corresponding to engagement in socially/ cognitively demanding activities.

Cognitive function: *Executive function* assessments included sub-scores of the digit span test (forward, backward and sequence),[3] language fluency (category and verbal) and part B of the Trail-making test (TMT).[4] Measures of *memory* included the Rey-Osterrieth Complex Figure (RCF; immediate recall, delayed recall and recognition)[5] and Hopkins Verbal Learning Test Revised (HVLT-R; total recall, delayed recall and recognition).[6] *Processing speed* was assessed by part A of the TMT,[4] digit coding[3] and simple reaction time, choice reaction time, simple movement and movement time[7] derived from the Cambridge Neuropsychological Test Automated Battery Reaction Time touchscreen task (CANTAB RTI; CANTABeclipse 5.0; Cambridge Cognition Ltd). For the CANTAB and TMT measures, scores were multiplied by -1 so that higher scores indicated better performance.

© 2020 Anatürk M et al. *JAMA Network Open.*

Demographic and scanner-related variables: Age was measured either at Phase 5 (for the trajectory analyses) or at the time of scan (neuroimaging analyses). Sex and education level were evaluated at the time of scan. Educational level was assessed on a 5-point scale: (1) no qualifications, (2) O-levels or equivalent (at 16 years), (3) A-levels, college certificate or professional qualification (at 18+ years), (4) degree (BSc, BA), (5) higher degree (MA, MSc, PhD). As a new scanner was introduced mid-way through data collection, the model of scanner is included as a further covariate. To account for motion-related variance, we included an index of mean head motion during the acquisition of functional images. This is a similar approach to other neuroimaging studies.[8,9] In our study, relative mean displacement obtained from McFLIRT was specifically used, due to its sensitive to changes in head position between consecutive volumes (F. Alfaro-Almagro 2019, personal communication, 11th May 2019).

MRI data acquisition: High resolution T_1-weighted images were acquired using a three-dimensional rapid gradient echo sequence with a repetition time of 2530ms, echo time of 1.79/3.65/5.51/7.37 ms (Prisma: 3.97 ms), flip angle of 7°, 256 mm field of view and 1.0 mm isotropic voxels. Diffusion-weighted images were collected using echo-planar imaging, with 60 diffusion weighted directions (b-value 1500s/mm^2), five non-diffusion weighted images (b-value 0s/mm^2) and a single b0 volume collected in the reversed phase encoded direction. The repetition time was 8900 ms with an echo time of 91.2 ms (Prisma: 91 ms), a field of view of 192 mm, with 2.0 mm isotropic voxels. Resting-state functional images were acquired using multiband echo-planar imaging, with a repetition time of 1300 ms, echo time of 40 ms, flip angle of 66°, field of view of 212 mm and 2.0 mm isotropic voxels. Fluid attenuated inversion recovery (FLAIR) images were used to identify white matter hyperintensities (repetition time = 9000ms, echo time = 73 ms, field of view = 220 ms, anisotropic voxels = 0.9 x 0.9 x 3.0 mm^3, Prisma: 0.4 x 0.4 x 3.0 mm^3).

© 2020 Anatürk M et al. *JAMA Network Open.*

MRI data pre-processing: FSL-VBM[10] was used to examine the associations between activities and voxel-wise measure of grey matter. The raw T_1-weighted images were first reoriented to a standard MNI template, bias field corrected, and registered to the MNI template using linear[11] and non-linear registration[12]. Brain tissue was then segmented into GM, WM and cerebrospinal fluid (CSF) using FMRIB's Automated Segmentation Tool (FAST)[13] and global volumetric measures of these tissues were extracted. Global GM and WM volumes were adjusted for total intracranial volume. T1-weighted images were brain extracted, grey matter segmented and then registered to the MNI 152 standard space with non-linear registration.[12] These images were averaged and flipped along the x-axis to produce a symmetrical, study-specific grey matter template. All native grey matter images were non-linearly registered to the grey matter template and modulated to correct for local expansions and contractions. The resulting images were smoothed with an isotropic Gaussian kernel with a sigma of 3 mm.

The Tract-Based Spatial Statistics (TBSS)[14] pipeline was used in the analysis of white matter microstructure. For the diffusion-weighted images, FSL's *topup* was first applied in order to estimate the susceptibility induced off-resonance field using the b0 scans.[15] Eddy was then used to correct for distortions attributed to motion and eddy currents.[16] If a given slice was >3 standard deviations from the Gaussian process predicted slice these were labelled as outliers and replaced. Volumes with >10 'outlier' slices were excluded. Participants with more than 5 volumes missing from their scans were excluded from the analysis. Diffusion-weighted scans were subsequently submitted to DTIFIT, which uses a diffusion tensor model to derive spatial maps of fractional anisotropy (FA), axial diffusivity (AD), mean diffusivity (MD) and radial diffusivity (RD) for each individual. The resulting images were brain extracted with FSl's Brain Extraction Tool.[17] Each individual's FA, AD, RD and MD images was then non-linearly registered into standard MNI space using

© 2020 Anatürk M et al. *JAMA Network Open.*

FMRIB58_FA as the target image. Subsequently, FA AD, RD and MD values were projected onto a study-specific mean FA tract skeleton, to derive skeletons for every participant. The averaged skeletons were intensity thresholded (= 0.2), in order to represent shared tracts across the entire sample. Mean FA, AD, RD and MD was also calculated for each participant, by averaging over these values across the entire white matter skeleton. We extracted white matter lesions (WML) using Brain Intensity AbNormality Classification Algorithm (BIANCA)[18]. All WML segmentations were visually inspected, excluding those that were identified as inaccurate.

Resting-state functional MRI (fMRI) images underwent the following pre-processing steps: motion correction, brain extraction, high-pass temporal filtering (cut-off = 100 sec), field map corrections; performed using FSL Multivariate Exploratory Linear Optimized Decomposition into Independent Components (MELODIC).[19] Artefactual components attributed to non-neuronal fluctuations were removed with single-subject ICA and FMRIB's ICA-based X-noiseifier (FIX).[20,21] The training data for FIX were from the WhII_MB6.RData trained-weights file (available at http://www.fmrib.ox.ac.uk/datasets/FIX-training/), consisting of manually labelled data from 25 participants. After pre-processing and cleaning, all resting-state images were registered to the individual's structural scan and standard space images using FNIRT. The images were then spatially smoothed using an isotropic Gaussian kernel of 6 mm full width at half maximum (FWHM). In order to create group-level spatial maps, MELODIC group-ICA was performed with 25 components. These spatial maps were created from all Whitehall II imaging sub-study participants with usable resting-state images without any neurological diseases or structural abnormalities (n = 678). MA and SS categorised the derived components as signal or noise. Dual regression was then used to extract subject-specific maps for each of the signal components. For the present analyses, only components representing the DMN, ECN and FPN are considered (n = 6, Figure S3).

© 2020 Anatürk M et al. *JAMA Network Open.*

Missing data: Instead of excluding respondents who had omitted a single item on the activity questionnaire, we used the weighted mean score from all items available at each time point. Weighted means for each time point were used to reduce the bias introduced by the one missing item on the overall summary score.

Full Information Maximum Likelihood was employed to address situations where an entire questionnaire was missing from a participant at a particular phase (e.g. due to non-attendance on the assessment day). This method uses all available information to estimate population parameters, which produces less biased estimates relative to common deletion (i.e. pair-wise or list-wise) and mean imputation approaches to addressing missing data.[22–24]

Trajectory analyses: We assessed the fit of the LGCM models based on the Akaike Information Criterion (AIC), Bayesian Information Criterion (BIC), and Sample-Size Adjusted BIC (SSA-BIC), Tucker-Lewis Index (TLI), Comparative Fit Index (CFI)[25] and Root Mean Square Error of Approximation (RMSEA).[26] Following Grimm et al.,[27] we considered adequate fit as a combination of the following: TLI ≥ 0.95, CFI ≥ 0.95[28] and a RMSEA ≥ 0.10.[29] AIC, BIC, and SSA-BIC were also used to compare models, with lower values indicating better fit.[27] As large sample sizes can bias the likelihood ratio chi-square towards rejecting even well-fitting models,[30] this statistic was not considered in our comparisons. Among the LCGMs, the model with best fit was identified as having the lowest AIC, BIC and SSA-BIC values, entropy values ≥ 0.8 and a p-value < 0.05 for the Lo-Mendel-Rubin likelihood ratio test and Bootstrap likelihood ratio test. A minimum of 5% of participants within each class was also considered essential for model selection.[31]

For both the LGCM and LCGM analyses, time scores were entered as the mean years since the baseline assessment (i.e. 0, 6, 9, 11 and 15 years since Phase 5), dividing by either 10 for LGCMs[32] or 100 for LCGMs[33] to aid model convergence. Further,

© 2020 Anatürk M et al. *JAMA Network Open.*

variances of the observed variables (i.e. activity measures) were constrained to be equal over time. LGCM analyses and LCGM were conducted in MPLUS (version 8). All LCGM were run with at least 100 sets of random sets of starting values, 10 optimizations and 10 iterations. If convergence was not achieved, the number of random starts optimizations and iterations were increased, as done in previous publications.[34] Persistent issues in model convergence or estimation are reported as experiencing "convergence problems".

Interpretation of parameters: As the quadratic growth curve model was deemed the best fit, parameters describing each individual's trajectories were extracted,[35] i.e. intercepts, linear and quadratic slopes (herein, referred to as intercept, linear and quadratic coefficients). To reduce collinearity between the linear and quadratic coefficients, time scores were centred at the middle time point (Phase 8; L. Müthen 2018, personal communication, 27th July 2018). Intercepts, therefore, reflected the estimated mean at Phase 8, with linear coefficients representing the annual rate of change in activities at this time point.[27] The quadratic coefficients, on the other hand, reflected *change* in activities across time, otherwise interpreted as the acceleration or deceleration of change in activity levels.[27]

As discussed in the main text, multicollinearity was detected between intercepts and quadratic coefficients for the analyses examining cognitive activity trajectories. As including both variables in a model may lead to biased results, their relationship with cognitive and MRI outcomes were examined separately, while adjusting for linear coefficients (in addition to age and other co-variates described in main text: Statistical analyses). The rationale here was that linear coefficients were one of two estimates of change in activities over time, with change representing an important co-variate when considering the relationship between activity level (i.e. intercepts) and brain/cognitive markers. For example, individuals who decline in activities at a faster rate over the study period, may report lower activity levels measured at a given time point. Given that the quadratic and linear coefficients are intricately

© 2020 Anatürk M et al. *JAMA Network Open.*

related (i.e. quadratic coefficients are accelerations/decelerations the rate of change,[27] i.e. linear coefficient), we also considered that the latter to be an essential co-variate to determine the independent contribution of quadratic change to the outcomes of interest.

Voxel-based analyses: For the voxel-wise analyses, we report clusters that survived family-wise error corrections for multiple comparisons across space and consisted of at least 10 voxels. An FDR q-value < 0.05 was considered significant.

Post hoc interaction analyses: The sample was stratified into two groups based on MoCA performance: high MoCA = ≥ 26; low MoCA = < 26. Interaction terms were added to the linear regression models to evaluate whether any of the significant associations detected in the main analysis, may be moderated by cognitive status (healthy vs. impaired). The interaction terms consisted of: (a) Intercept coefficients of cognitive activities x MoCA status, (b) Linear coefficients of cognitive activities x MoCA status, (c) Quadratic coefficients of cognitive activities x MoCA status, (d) Intercepts coefficients of social activities x MoCA status, (b) Linear coefficients of social activities x MoCA status, (c) Quadratic coefficients of social activities x MoCA status.

© 2020 Anatürk M et al. *JAMA Network Open.*

eResults. Supplementary Results

Comparisons of included and excluded participants: On average, participants included in the analyses were younger ($p = 0.018$), more educated ($p = 0.031$) and achieved higher MoCA scores ($p < 0.001$) relative to excluded participants. There were no differences in the proportion of females between these groups (For results, see eTable 6).

© 2020 Anatürk M et al. *JAMA Network Open.*

eFigure 1. A timeline of the study phases and variables included in the present analyses.

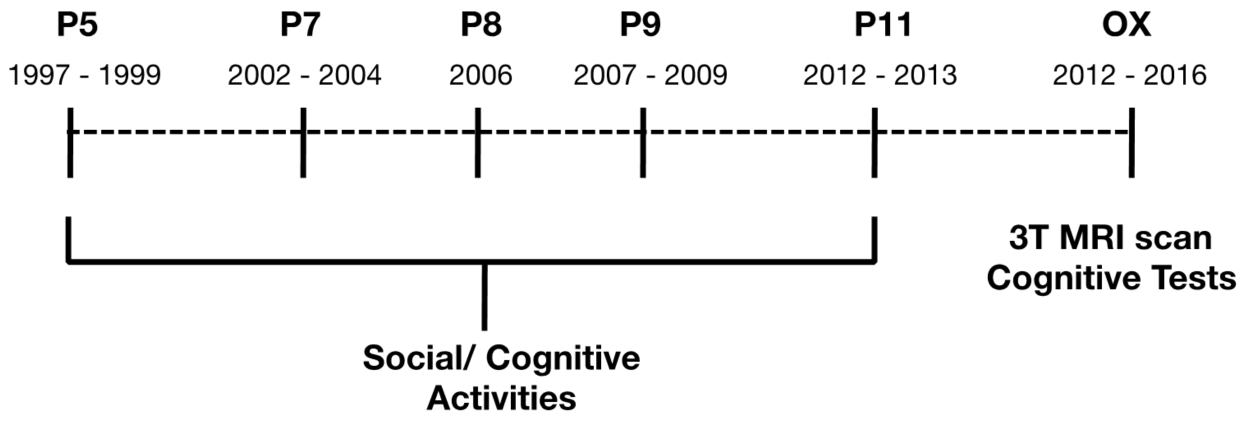

Abbreviations: MRI = Magnetic resonance imaging; OX = Oxford scanning visit; P = Phase.

© 2020 Anatürk M et al. *JAMA Network Open.*

eFigure 2. Flowchart of participant selection and exclusion.

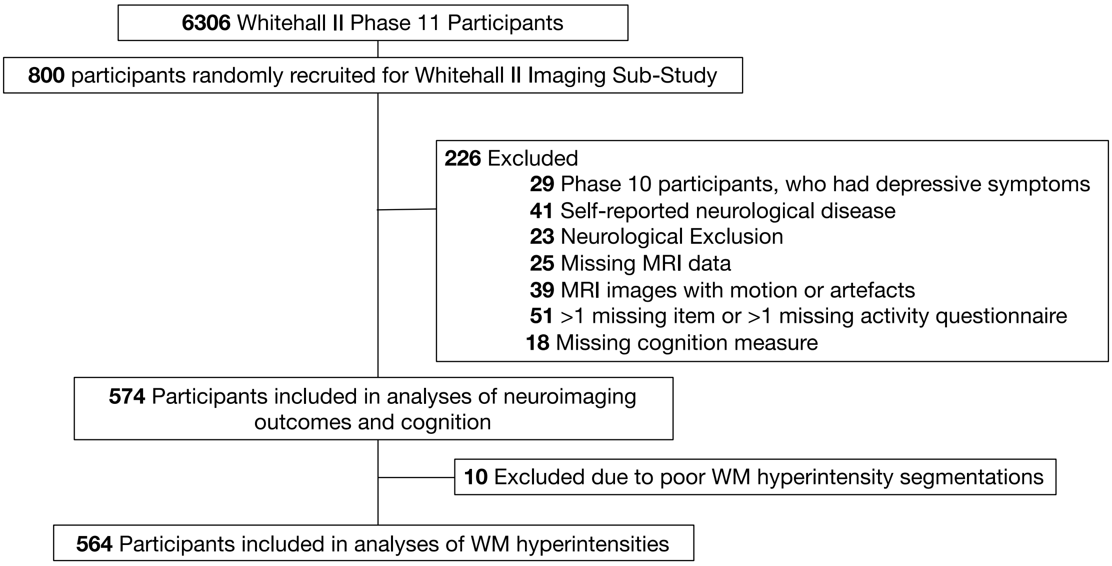

Abbreviations: WM = White matter.

© 2020 Anatürk M et al. *JAMA Network Open.*

eFigure 3. ICA components representing the executive control, default model and fronto-parietal networks.

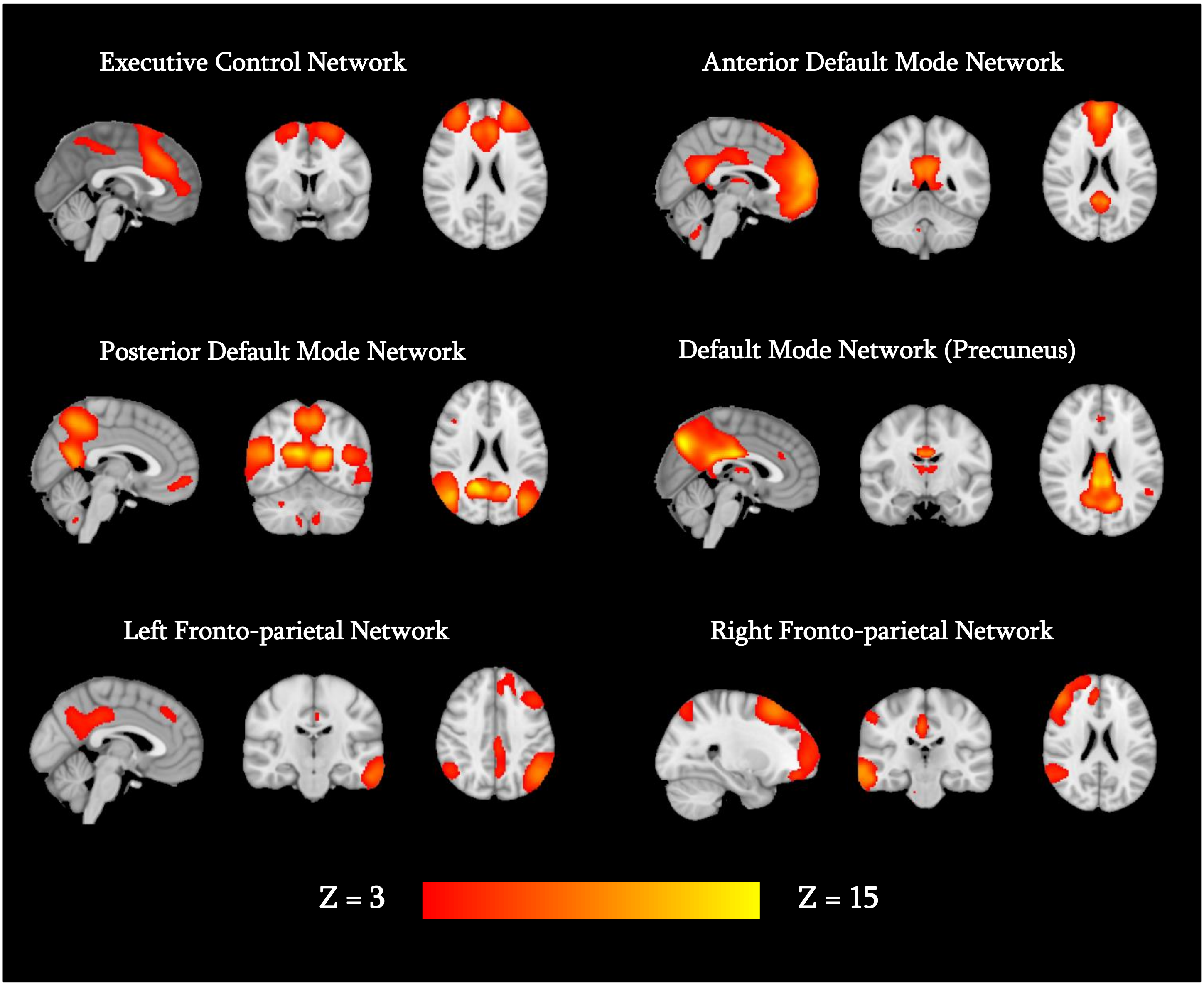

© 2020 Anatürk M et al. *JAMA Network Open.*

eTable 1. Social and cognitive demand ratings for each of the items of the WHII activity questionnaire.

	Social Demand Rating	Cognitive Demand Rating
Q1: Religious activities/ observance.	High	Medium
Q2: Positions of office, school governor, councillor ect.	High	High
Q3: Involvement in clubs and organisations, voluntary or official. (Phase 5) *Or* Voluntary work (Phase 7 onwards).	High	Medium
Q4: Courses and educational/ evening classes.	Medium	High
Q5: Cultural visits to stately homes, galleries, theatres, cinema or live music events.	Medium	High
Q6: Social indoor games, cards, bingo, chess, ect.	High	High
Q7: Visiting friends and relatives.	High	Medium
Q8: Going to pubs and social clubs.	High	Medium
Q9: Individual occupations, e.g. reading, listening to music	Low	High
Q10: Household tasks, e.g. DIY, maintenance, decorating.	Low	Low
Q11: Practical activities, making things with your hands, e.g. pottery, drawing, ect.	Low	Medium
Q12: Gardening.	Low	Low
Q13: Using a home computer for leisure.	Low	High
**Note:** Items marked as having low social/cognitive demand were excluded from the composite activity measures.		

© 2020 Anatürk M et al. *JAMA Network Open.*

eTable 2. A description of each test within the Whitehall II cognitive battery. For further details, please see Filippini et al. (2014).

Cognitive test	Description
MoCA (Nasreddine et al., 2005)	The MoCA is a 10-minute cognitive screening test that assesses multiple domains, including visuospatial abilities, executive function and language. This test integrates a range of sub-tests, such as the naming of low-familiarity animals, short-term memory recall task, a clock-drawing task, a three-dimension cube copy task, alphanumeric trail making, phonemic fluency task, verbal abstraction task, digit forward and backward and orientation. MoCA scores ranges from 0 – 30, with higher scores reflecting better overall cognition. An additional one point is given to an individual with less than 12 years of education. In a clinical setting, scores below 26 may indicate cognitive impairment (Nasreddine et al., 2005).
Digit span (Wechsler, 2008)	In this task, a trained psychology graduate read out a series of numbers. Participants were either required to recall the numbers in the same order (digit forward), in reverse order (digit backward) or from smallest to largest number (digit sequence). The outcome was the maximum number of digits correctly recalled, under each condition.
Digit Coding (Wechsler, 2008)	Participants were presented with a key that contained a series of numbers, with a unique symbol associated with each number. In a grid containing just numbers, the main task was to draw the correct symbol paired with each number (as stated in the key), within a 2-minute period. The outcome was the total number of correct digit symbol matches.
Language fluency (adapted from ACE-III; Hsieh et al. 2013)	This task required individuals to list as many words as possible starting with the letter 'S' (verbal fluency) or name as many animals as possible (category fluency) within a 60 second time frame. The outcome was the number of words recalled for each type of language fluency.
TMT A and B (Reitan, 1958)	For the trail making tasks, participants were instructed to connect a series of distributed circles on a page consisting of 25 numbers (TMT A) or numbers and letters (TMT B) as quickly and as accurately as possible. The outcome was the time taken to correctly complete the trail (seconds).

© 2020 Anatürk M et al. *JAMA Network Open.*

RCF (Osterrieth, 1944)	Participants were presented with a complex geometric diagram that they were initially asked to copy. The image was then removed, with individuals immediately instructed to redraw the diagram, this time, from memory (outcome 1: immediate recall score). After a delay, participants were required to once more draw the image from memory (outcome 2: delayed recall score). In the final section of the RCF, participants were presented with several geometric shapes, and asked whether they formed part of the original complex diagram (outcome 3: recognition score).
HVLT-R (Brandt, 1991)	This test required individuals to learn a list of 12 words (drawn from three semantic categories, such as precious gems or vegetables), through three learning trials. A delayed recall task was then administered with a delay of 20-25 minutes, which was followed by subsequent recognition task. For the recognition task, individuals were presented with 24 words and required to identify whether a given word had been in the original list of words to learn (12 were correct). This task therefore provided a measure of delayed recall, recognition and total recall.
CANTAB RTI (CANTABeclipse 5.0; Cambridge Cognition Ltd)	This computerized test (delivered on a touchscreen table) consisted of a simple and choice reaction time task. The simple reaction time task instructed individuals to maintain their finger on a button on the screen until a yellow dot appeared, with the task being to move their finger to the yellow dot as quickly as possible. Under this condition, the yellow dot had only one possible location that it could appear in. In the choice reaction time task, the yellow dot could appear in one of five locations. The outcomes were reaction time (i.e. time to release the button) and movement time (i.e. time taken to touch the yellow dot after releasing the button) in each task.
**Abbreviations-** ACE-III = Addenbrooke's Cognitive Examination Revised; CANTAB RTI = Cambridge Neuropsychological Test Automated Battery Reaction Time touchscreen task; HVLT-R = Hopkins Verbal Learning Test Revised; RCF = Rey-Osterrieth Complex Figure; RDI = Recognition discrimination index; TMT = Trail making test.	

© 2020 Anatürk M et al. *JAMA Network Open.*

eTable 3. MPLUS syntax used to generate a quadratic LGCM. Adapted from Stride (2016)[37] and Jung and Wickrama (2008).[34]

**Title:**	**Quadratic growth curve model for social activities**
**Data:**	file = SOC_n574.dat;
**Variable:**	names are ID age sex edu<br>sa_p5 sa_p7 sa_p8 sa_p9 sa_p11;<br>IDvar = ID;<br>usevar = sa_p5-sa_p11;<br>missing = all (-999);
**Analysis:**	type = missing H1;<br>estimator = MLR;
**Model:**	i s q | sa_p5@0 sa_p7@0.6 sa_p8@0.9 sa_p9@1.1 sa_p11@1.5;<br>sa_p5 (a);<br>sa_p7 (a);<br>sa_p8 (a);<br>sa_p9 (a);<br>sa_p11 (a);
**Output:**	sampstat standardized tech1;
**Plot:**	Series = sa_p5-sa_p11 (s);<br>TYPE = PLOT3;
**SAVEDATA:**	FILE IS Soc_isq_Fscores;<br>save = FSCORES;

© 2020 Anatürk M et al. *JAMA Network Open.*

eTable 4. MPLUS syntax used to produce a 3-class linear LCGM. Adapted from Stride (2016)[37] and Jung and Wickrama (2008).[34]

**Title:**	**Quadratic growth curve model for social activities**
**Data:**	file = SOC_n574.dat;
**Variable:**	names are ID age sex edu<br>sa_p5 sa_p7 sa_p8 sa_p9 sa_p11;<br>IDvar = ID;<br>usevar = sa_p5-sa_p11;<br>CLASSES = c(3);<br>missing = all (-999);
**Analysis:**	type = MIXTURE missing;<br>STARTS = 100 10;<br>STITERATIONS = 10;<br>! LRTSTARTS = 0 0 700 80;<br>estimator = MLR;
**Model:**	%OVERALL%<br>i s | sa_p5@0 sa_p7@0.06 sa_p8@0.09 sa_p9@0.11 sa_p11@0.15;<br>i-s@0;<br>sa_p5 (a);<br>sa_p7 (a);<br>sa_p8 (a);<br>sa_p9 (a);<br>sa_p11 (a);
**Output:**	sampstat standardized tech1<br>TECH8 TECH11 TECH14;
**Plot:**	Series = sa_p5-sa_p11 (s);<br>TYPE = PLOT3;
**SAVEDATA:**	FILE IS Soc_3class_Linear_CProbabilities;<br>save = cprobabilities;

© 2020 Anatürk M et al. *JAMA Network Open.*

eTable 5. SPSS syntax used for a linear regression with the intercept, linear and quadratic coefficients of social activity as the predictors of interest and FA as the outcome variable.

```
REGRESSION
  /MISSING LISTWISE
  /STATISTICS COEFF OUTS R ANOVA COLLIN TOL CHANGE
  /CRITERIA=PIN(.05) POUT(.10)
  /NOORIGIN
  /DEPENDENT FA
  /METHOD=ENTER  I_SA S_SA Q_SA age sex edu scanner head_motion
  /PARTIALPLOT ALL
  /SCATTERPLOT= (*ZRESID ,*ZPRED)
  /RESIDUALS DURBIN HISTOGRAM(ZRESID) NORMPROB(ZRESID)
  /CASEWISE PLOT(ZRESID) OUTLIERS(3).
```

© 2020 Anatürk M et al. *JAMA Network Open.*

eTable 6. Comparisons of included and excluded participants.

	Included	Excluded	Test-statistic	*p*
N	574	226		
t-tests			t	
Age at scan	69.57 ± 4.93	70.6 ± 5.72	2.370	0.018
MoCA, median (IQR)[1]	28 (26 – 29)	27 (25 – 28)	U = 4.112	< 0.001
Education	3.54 ± 1.06	3.35 ± 1.11	-2.166	0.031
Chi-squared test			$\chi 2$	
N of females (%)	106 (18.5%)	46 (20.4%)	0.375	0.549

Note. Mean ± SD is reported unless otherwise stated.
[1]Due to skewed data, Mann-Whitney U test was performed.
Abbreviations: MoCA = Montreal Cognitive Assessment; N = Number.

© 2020 Anatürk M et al. *JAMA Network Open.*

eTable 7. Model fit indices for all of the unconditional latent growth curve models assessed.

	AIC	BIC	SSA-BIC	CFI	TLI	RMSEA (C.I.)
Cognitive Activity						
Intercept only	2274.868	2287.925	2278.402	0.096	0.468	0.325 (0.309 - 0.342)
Linear	1620.921	1647.037	1627.989	0.641	0.743	0.226 (0.208 - 0.245)
**Quadratic**	**1229.384**	**1272.91**	**1241.164**	**0.967**	**0.967**	**0.081 (0.058 - 0.104)**
Social Activity						
Intercept only	2609.447	2622.505	2612.981	0.736	0.845	0.189 (0.173 - 0.206)
Linear	2445.187	2471.303	2452.256	0.853	0.895	0.155 (0.137 - 0.174)
**Quadratic**	**2371.436**	**2414.962**	**2383.216**	**0.909**	**0.909**	**0.145 (0.123 - 0.167)**

Abbreviations: AIC = Akaike Information Criterion; BIC = Bayesian Information Criterion; CFI = Comparative fit index; C.I. = Confidence Intervals; RMSEA = Root Mean Square Error of Approximation; SSA- BIC = Sample size adjusted Bayesian Information Criterion; TLI = Tucker-Lewis Index.

© 2020 Anatürk M et al. *JAMA Network Open.*

eTable 8. As the quadratic latent growth curve model best described the pattern of growth in the sample, the table demonstrates the fit indices for all of the unconditional quadratic latent class growth models. The results for the intercept only, linear and conditional models can be requested from the authors.

	AIC	BIC	SSA-BIC	Entropy	Class 1	Class 2	Class 3	Class 4	Class 5	Class 6	LMR-LRT p	BLRT p
Social Activity												
2 classes	2845.784	2880.605	2855.209	0.86	347	227					< 0.001	< 0.001
3 classes	2575.48	2627.712	2589.617	0.835	85	224	265				0.008	< 0.001
4 classes	2472.393	2542.035	2491.242	0.774	154	53	150	217			0.23	< 0.001
5 classes	2425.45	2512.503	2449.011	0.775	142	39	212	147	34		0.348	< 0.001
6 classes	2399.544	2504.007	2427.817	0.799	203	146	31	44	11	139	0.3531	< 0.001
Cognitive Activity												
2 classes	1688.89	1723.711	1698.315	0.802	339	235					< 0.001	< 0.001
3 classes	1428.067	1480.298	1442.203	0.8	113	317	144				0.0022	< 0.001
4 classes	1349.468	1419.11	1368.317	0.759	78	69	252	175			0.0216	< 0.001
5 classes	1308.241	1395.293	1331.802	0.73	62	73	87	232	120		0.0091	< 0.001
6 classes	*No convergence*											

Abbreviations: AIC = Akaike Information Criterion; BIC = Bayesian Information Criterion; BLRT = Bootstrap Likelihood Ratio Test; LMR-LRT = Lo-Mendel-Rubin Likelihood Ratio Test; SSA-BIC = Sample Size Adjusted Bayesian Information Criterion

© 2020 Anatürk M et al. *JAMA Network Open.*

eTable 9. Regression coefficients for cognitive activities.

Linear regression analyses were used to investigate whether intercepts, linear and quadratic coefficients of cognitive activities associate with cognitive and MRI measures. Analyses are adjusted for age, sex and educational level with further adjustments made for scanner model in the regressions related to MRI outcomes. Due to multicollinearity between intercepts and quadratic coefficients, no mutual adjustments were made. Unstandardized beta estimates (B), their standard errors (SE), standardized beta estimates (β) and p-values are reported.

Dependent Variable	Predictors (CA)	B	SE	β	p-value
Cognitive function					
**Global cognition**	**Intercept**	**0.955**	**0.285**	**0.140**	**0.001**
	Linear Coefficient[a]	-0.330	0.902	-0.015	0.715
	Linear Coefficient[b]	-1.075	0.944	-0.048	0.255
	**Quadratic Coefficient**	**-1.382**	**0.492**	**-0.122**	**0.005**
**Executive function**	**Intercept**	**1.831**	**0.499**	**0.148**	**<0.001*****
	Linear Coefficient[a]	0.818	1.579	0.02	0.604
	Linear Coefficient[b]	-0.374	1.657	-0.009	0.822
	**Quadratic Coefficient**	**-2.219**	**0.865**	**-0.107**	**0.011**
**Memory**	**Intercept**	**1.394**	**0.550**	**0.106**	**0.012**
	Linear Coefficient[a]	0.937	1.741	0.022	0.59
	Linear Coefficient[b]	-0.335	1.817	-0.008	0.854
	**Quadratic Coefficient**	**-2.355**	**0.948**	**-0.107**	**0.013**
**Processing speed**	**Intercept**	**1.514**	**0.528**	**0.118**	**0.004**
	Linear Coefficient[a]	0.28	1.67	0.007	0.867
	Linear Coefficient[b]	-0.545	1.751	-0.013	0.756
	Quadratic Coefficient	-1.543	0.913	-0.072	0.092
Brain structure					
**Global grey matter volume (% of ICV)**	Intercept	0.416	0.226	0.066	0.066
	Linear Coefficient[a]	1.076	0.717	0.053	0.134
	Linear Coefficient[b]	0.582	0.748	0.029	0.437
	**Quadratic Coefficient**	**-0.910**	**0.388**	**-0.087**	**0.019**
**Global white matter volume (% of ICV)**	Intercept	0.069	0.223	0.009	0.757
	Linear Coefficient[a]	0.555	0.708	0.023	0.434
	Linear Coefficient[b]	0.664	0.739	0.027	0.37
	Quadratic Coefficient	0.193	0.384	0.015	0.615
**Global white matter lesions (% of ICV)[c]**	Intercept	-0.025	0.026	-0.037	0.343
	Linear Coefficient[a]	-0.099	0.083	-0.045	0.234
	Linear Coefficient[b]	-0.09	0.087	-0.041	0.299
	Quadratic Coefficient	0.018	0.045	0.016	0.693
**FA**	Intercept	0	0.002	-0.005	0.894
	Linear Coefficient[a]	0.006	0.007	0.03	0.427
	Linear Coefficient[b]	0.007	0.008	0.036	0.358
	Quadratic Coefficient	0.002	0.004	0.022	0.578
**AD (x10^3)**	Intercept	0.002	0.003	0.029	0.462
	Linear Coefficient[a]	-0.001	0.01	-0.004	0.917
	Linear Coefficient[b]	-0.005	0.01	-0.02	0.619
	Quadratic Coefficient	-0.007	0.005	-0.057	0.162
**MD (x10^3)**	Intercept	0.002	0.003	0.019	0.626
	Linear Coefficient[a]	-0.006	0.01	-0.021	0.573

© 2020 Anatürk M et al. *JAMA Network Open.*

	Linear Coefficient[b]	-0.009	0.01	-0.033	0.395
	Quadratic Coefficient	-0.006	0.005	-0.043	0.275
**RD (x10³)**	Intercept	0.001	0.003	0.013	0.728
	Linear Coefficient[a]	-0.008	0.011	-0.028	0.458
	Linear Coefficient[b]	-0.011	0.011	-0.038	0.334
	Quadratic Coefficient	-0.005	0.006	-0.035	0.372
Resting-state functional connectivity*					
**Anterior DMN**	Intercept	0.594	0.744	0.032	0.425
	Linear Coefficient[a]	0.501	2.362	0.008	0.832
	Linear Coefficient[b]	-0.501	2.463	-0.008	0.839
	Quadratic Coefficient	-1.834	1.28	-0.059	0.153
**DMN (Precuneus)**	Intercept	-0.563	0.687	-0.033	0.412
	Linear Coefficient[a]	0.527	2.18	0.009	0.809
	Linear Coefficient[b]	0.579	2.278	0.01	0.8
	Quadratic Coefficient	0.118	1.184	0.004	0.92
**Posterior DMN**	Intercept	-0.208	0.472	-0.018	0.66
	Linear Coefficient[a]	0.835	1.498	0.023	0.577
	Linear Coefficient[b]	0.702	1.564	0.019	0.654
	Quadratic Coefficient	-0.232	0.813	-0.012	0.776
**Left FPN**	Intercept	-0.213	0.653	-0.013	0.744
	Linear Coefficient[a]	1.372	2.073	0.026	0.508
	Linear Coefficient[b]	1.434	2.165	0.027	0.508
	Quadratic Coefficient	0.121	1.125	0.004	0.915
**Right FPN**	Intercept	-0.328	0.579	-0.024	0.571
	Linear Coefficient[a]	1.004	1.839	0.022	0.585
	Linear Coefficient[b]	1.188	1.921	0.026	0.537
	Quadratic Coefficient	0.347	0.998	0.015	0.729
**ECN**	Intercept	0.030	0.529	0.002	0.955
	Linear Coefficient[a]	-0.597	1.681	-0.014	0.722
	Linear Coefficient[b]	-0.888	1.755	-0.021	0.613
	Quadratic Coefficient	-0.526	0.912	-0.024	0.564

Bold = p <0.05; ***** = survived FDR corrections.
[a] Adjusted for intercepts;
[b] Adjusted for quadratic coefficients
[c] Log transformed.
Abbreviations: AD = Axial diffusivity; B = unstandardized beta coefficient; β = standardized beta coefficient; CA = Cognitive activity; CSF = Cerebrospinal fluid; DMN = Default mode network; ECN = Executive control network; FA = Fractional anisotropy; FPN = Fronto-parietal network; GM = Grey matter; ICV = Intracranial volume; MD = Mean diffusivity; MoCA = Montreal Cognitive Assessment; MRI = Magnetic resonance imaging; SE = Standard error; RD = Radial diffusivity; WM = White matter; WM = White matter lesions.

© 2020 Anatürk M et al. *JAMA Network Open.*

eTable 10. Regression coefficients for social activities.

Results of linear regression analyses evaluating whether intercepts, linear and quadratic coefficients of social activities associated with cognitive and brain markers, adjusted for age, sex and educational level. The effects of scanner model and head motion are also accounted for in the analyses of MRI metrics. Unstandardized beta estimates (B), their standard errors (SE), standardized beta estimates (β) and p-values are reported.

Dependent variable	Predictors (SA)	B	SE	β	p-value
Cognition					
Global cognition	Intercept	0.219	0.302	0.042	0.491
	Coefficient	-0.374	0.703	-0.036	0.676
	Quadratic	0.369	0.59	0.036	0.514
Executive function	**Intercept**	**1.695**	**0.525**	**0.179**	**0.001*****
	Coefficient	-1.371	1.221	-0.053	0.262
	**Quadratic**	**2.542**	**1.026**	**0.135**	**0.014**
Memory	Intercept	-0.229	0.581	-0.023	0.694
	Coefficient	0.139	1.351	0.005	0.918
	Quadratic	-0.97	1.135	-0.048	0.393
Processing speed	Intercept	0.907	0.555	0.093	0.103
	Coefficient	0.803	1.291	0.03	0.535
	Quadratic	1.43	1.085	0.073	0.188
Brain structure					
Global GM volume (% of ICV)	Intercept	-0.047	0.238	-0.01	0.842
	Coefficient	0.317	0.554	0.024	0.568
	Quadratic	-0.629	0.464	-0.066	0.176
Global WM volume (% of ICV)	Intercept	-0.136	0.234	-0.024	0.561
	Coefficient	0.504	0.545	0.032	0.356
	Quadratic	-0.033	0.457	-0.003	0.942
Global WML volume (% of ICV)[a]	Intercept	-0.003	0.028	-0.006	0.91
	Coefficient	-0.038	0.064	-0.027	0.551
	Quadratic	0.036	0.054	0.036	0.5
FA	Intercept	0.000	0.002	-0.008	0.883
	Coefficient	0.001	0.006	0.004	0.924
	Quadratic	0.002	0.005	0.025	0.631
AD (x10^3)	Intercept	-0.001	0.003	-0.009	0.863
	Coefficient	-7.612E-05	0.008	0	0.992
	Quadratic	-0.008	0.006	-0.071	0.181
MD (x10^3)	Intercept	5.741E-05	0.003	0.001	0.986
	Coefficient	0.000	0.008	-0.003	0.949
	Quadratic	-0.006	0.006	-0.051	0.327
RD (x10^3)	Intercept	0.000	0.004	0.005	0.918
	Coefficient	-0.001	0.008	-0.004	0.933
	Quadratic	-0.005	0.007	-0.039	0.445
Resting-state functional connectivity					
Anterior DMN	Intercept	0.122	0.778	0.009	0.875
	Coefficient	-0.315	1.813	-0.008	0.862
	Quadratic	-2.224	1.519	-0.078	0.144
DMN (Precuneus)	Intercept	-1.13	0.72	-0.087	0.117

© 2020 Anatürk M et al. *JAMA Network Open.*

	Coefficient	0.339	1.677	0.01	0.84
	Quadratic	-1.208	1.404	-0.046	0.39
Posterior DMN	Intercept	-0.623	0.495	-0.073	0.208
	Coefficient	0.74	1.153	0.032	0.521
	Quadratic	-1.037	0.965	-0.061	0.283
Left FPN	Intercept	0.255	0.686	0.021	0.71
	Coefficient	-0.645	1.597	-0.019	0.687
	Quadratic	-0.444	1.338	-0.018	0.74
Right FPN	Intercept	-0.07	0.608	-0.007	0.908
	Coefficient	-0.112	1.417	-0.004	0.937
	Quadratic	-0.666	1.187	-0.031	0.575
ECN	Intercept	0.172	0.554	0.017	0.756
	Coefficient	-1.525	1.291	-0.055	0.238
	Quadratic	-0.590	1.082	-0.029	0.585

Bold = p <0.05; ******* survives FDR corrections.

[a]log-transformed due to skewed data.

Abbreviations: AD = Axial diffusivity; B = unstandardized beta coefficient; β = standardized beta coefficient; CSF = Cerebrospinal fluid; DMN = Default mode network; ECN = Executive control network; FA = Fractional anisotropy; FPN = Fronto-parietal network; GM = Grey matter; ICV = Intracranial volume; MD = Mean diffusivity; MoCA = Montreal Cognitive Assessment; MRI = Magnetic resonance imaging; SA = Social activity; SE = Standard error; RD = Radial diffusivity; WM = White matter; WML = White matter lesions.

© 2020 Anatürk M et al. *JAMA Network Open.*

eTable 11. Interactions between MoCA group (MoCA score < 26 = 471); MoCA score ≥ 26 = 103) and activity trajectories. Other variables in the model: intercept, linear and quadratic coefficients (unless multicollinearity detected), MoCA group, age, sex and educational level. Continuous variables were demeaned before the interaction term was created. Unstandardized beta estimates (B), their standard errors (SE), standardized beta estimates (β) and p-values are reported.

Interaction terms	B	SE	β	p-value
Executive function				
MoCA x Intercept coefficient of CA	-0.339	1.175	-0.012	0.773
MoCA x Linear coefficient of CA (a)	5.212	3.560	0.061	0.144
MoCA x Linear coefficient of CA (b)	5.872	3.744	0.068	0.117
MoCA x Quadratic coefficient of CA	0.861	1.999	0.019	0.667
MoCA x Intercept coefficient of SA	-1.027	1.283	-0.043	0.424
MoCA x Linear coefficient of SA	5.307	3.002	0.092	0.078
MoCA x Quadratic coefficient of SA	-2.053	2.458	-0.049	0.404
Memory				
**MoCA x Intercept coefficient of CA**	**3.828**	**1.259**	**0.128**	**0.002**
MoCA x Linear coefficient of CA (a)	2.284	3.817	0.025	0.55
MoCA x Linear coefficient of CA (b)	0.036	4.010	0.000	0.993
**MoCA x Quadratic coefficient of CA**	**-5.179**	**2.141**	**-0.106**	**0.016**
MoCA x Intercept coefficient of SA	1.445	1.385	0.057	0.297
MoCA x Linear coefficient of SA	5.201	3.242	0.085	0.109
MoCA x Quadratic coefficient of SA	-0.026	2.655	-0.001	0.992
Processing Speed				
MoCA x Intercept coefficient of CA	0.973	1.297	0.034	0.454
MoCA x Linear coefficient of CA (a)	-4.477	3.932	-0.051	0.255
MoCA x Linear coefficient of CA (b)	-5.337	4.132	-0.06	0.197
MoCA x Quadratic coefficient of CA	-2.170	2.206	-0.046	0.326
MoCA x Intercept coefficient of SA	-0.112	1.420	-0.005	0.937
MoCA x Linear coefficient of SA	2.323	3.324	0.039	0.485
MoCA x Quadratic coefficient of SA	-2.988	2.722	-0.069	0.273
**Abbreviations:** CA = Cognitive Activities; MoCA = Montreal Cognitive Assessment; SA = Social Activities.				

© 2020 Anatürk M et al. *JAMA Network Open.*

eTable 12. Social activity trajectories were negatively correlated with voxel-wise measures of functional connectivity involving the sensorimotor and temporo-parietal networks. Coordinates are provided in MNI space. Note that these results did not survive FDR corrections.

				**MNI coordinates**			
	**ICA Component**	**N. of voxels**	**P-value**	**x**	**y**	**z**	**Region**
Social activity (quadratic coefficients)	Sensorimotor network	306	0.01	38	-32	64	R Postcentral gyrus
Social activity (linear coefficients)	Temporo-parietal network	16	0.02	26	-72	8	R Intracalcarine cortex
**Abbreviations:** ICA = Independent Component Analysis; MNI = Montreal Neurological Institute; N = Number.							

© 2020 Anatürk M et al. *JAMA Network Open.*

eReferences

1. Anatürk, M., Demnitz, N., Ebmeier, K. P. & Sexton, C. E. A systematic review and meta-analysis of structural magnetic resonance imaging studies investigating cognitive and social activity levels in older adults. *Neurosci. Biobehav. Rev.* (2018). doi:10.1016/J.NEUBIOREV.2018.06.012
2. Manikandan, S. Measures of central tendency: The mean. *J. Pharmacol. Pharmacother.* **2**, 140–2 (2011).
3. Wechsler, D. *Wechsler Adult Intelligence Scale–Fourth Edition (WAIS–IV).* (The Psychological Corporation, 2008).
4. Reitan, R. M. Validity of the Trail Making Test as an Indicator of Organic Brain Damage. *Percept. Mot. Skills* **8**, 271–276 (1958).
5. Osterrieth, P. A. Le test de copie d'une figure complexe; contribution à l'étude de la perception et de la mémoire. *Arch. Psychol. (Geneve).* **30**, 206–356 (1944).
6. Brandt, J. The hopkins verbal learning test: Development of a new memory test with six equivalent forms. *Clin. Neuropsychol.* **5**, 125–142 (1991).
7. Sahakian, B. J. *et al.* Further analysis of the cognitive effects of tetrahydroaminoacridine (THA) in Alzheimer's disease: assessment of attentional and mnemonic function using CANTAB. *Psychopharmacology (Berl).* **110**, 395–401 (1993).
8. Miller, K. L. *et al.* Multimodal population brain imaging in the UK Biobank prospective epidemiological study. *Nat. Neurosci.* **19**, 1523–1536 (2016).
9. Heise, V. *et al.* Apolipoprotein E genotype, gender and age modulate connectivity of the hippocampus in healthy adults. *Neuroimage* **98**, 23–30 (2014).
10. Douaud, G. *et al.* Anatomically related grey and white matter abnormalities in adolescent-onset schizophrenia. *Brain* **130**, 2375–2386 (2007).
11. Jenkinson, M. & Smith, S. A global optimisation method for robust affine registration of brain images. *Med. Image Anal.* **5**, 143–56 (2001).
12. Andersson, J. L. R. *et al.* Non-Linear Registration aka Spatial Normalisation FMRIB Technial Report TR07JA2. (2007).
13. Zhang, Y., Brady, M. & Smith, S. Segmentation of brain MR images through a hidden Markov random field model and the expectation-maximization algorithm. *IEEE Trans. Med. Imaging* **20**, 45–57 (2001).
14. Smith, S. M. *et al.* Tract-based spatial statistics: Voxelwise analysis of multi-subject diffusion data. *Neuroimage* **31**, 1487–1505 (2006).
15. Andersson, J. L. R., Skare, S. & Ashburner, J. How to correct susceptibility distortions in spin-echo echo-planar images: application to diffusion tensor imaging. *Neuroimage* **20**, 870–888 (2003).
16. Andersson, J. L. R. & Sotiropoulos, S. N. An integrated approach to correction for off-resonance effects and subject movement in diffusion MR imaging. *Neuroimage* **125**, 1063–1078 (2016).
17. Smith, S. M. Fast robust automated brain extraction. *Hum. Brain Mapp.* **17**, 143–155 (2002).
18. Griffanti, L. *et al.* BIANCA (Brain Intensity AbNormality Classification Algorithm): A new tool for automated segmentation of white matter hyperintensities. *Neuroimage* **141**, 191–205 (2016).
19. Beckmann, C. F., DeLuca, M., Devlin, J. T. & Smith, S. M. Investigations into resting-state

© 2020 Anatürk M et al. *JAMA Network Open.*

connectivity using independent component analysis. *Philos. Trans. R. Soc. B Biol. Sci.* **360**, 1001–1013 (2005).
20. Griffanti, L. *et al.* ICA-based artefact removal and accelerated fMRI acquisition for improved resting state network imaging. *Neuroimage* **95**, 232–247 (2014).
21. Salimi-Khorshidi, G. *et al.* Automatic denoising of functional MRI data: Combining independent component analysis and hierarchical fusion of classifiers. *Neuroimage* **90**, 449–468 (2014).
22. Enders, C. & Bandalos, D. The Relative Performance of Full Information Maximum Likelihood Estimation for Missing Data in Structural Equation Models. *Struct. Equ. Model. A Multidiscip. J.* **8**, 430–457 (2001).
23. Wothke, W. Longitudinal and multigroup modeling with missing data. in *Modeling longitudinal and multilevel data: Practical issues, applied approaches, and specific examples.* 219-240,269-281 (Lawrence Erlbaum Associates Publishers, 2000).
24. Little, T. D., Jorgensen, T. D., Lang, K. M. & Moore, E. W. G. On the Joys of Missing Data. *J. Pediatr. Psychol.* **39**, 151–162 (2014).
25. Bentler, P. M. Comparative fit indexes in structural models. *Psychol. Bull.* **107**, 238–246 (1990).
26. Steiger, J. H. & Lind, J. C. Statistically based tests for the number of common factors. *Annu. Meet. Psychom. Soc.* **Iowa City,** (1980).
27. Grimm, K. J., Ram, N. & Hamagami, F. Nonlinear Growth Curves in Developmental Research. *Child Dev.* **82**, 1357–1371 (2011).
28. Hu, L. & Bentler, P. M. Cutoff criteria for fit indexes in covariance structure analysis: Conventional criteria versus new alternatives. *Struct. Equ. Model. A Multidiscip. J.* **6**, 1–55 (1999).
29. MacCallum, R. C., Browne, M. W. & Sugawara, H. M. Power analysis and determination of sample size for covariance structure modeling. *Psychol. Methods* **1**, 130–149 (1996).
30. Preacher, K. J., Wichman, A. L., MacCallum, R. C. & Briggs, N. E. *Latent growth curve modeling.* (SAGE, 2008).
31. Andruff, H., Carraro, N., Thompson, A., Gaudreau, P. & Louvet, B. Latent Class Growth Modelling: A Tutorial. *Tutor. Quant. Methods Psychol.* **5**, 11–24 (2009).
32. Rosenberg, M. *et al.* A latent growth curve model to estimate electronic screen use patterns amongst adolescents aged 10 to 17 years. *BMC Public Health* **18**, 332 (2018).
33. Berlin, K. S., Parra, G. R. & Williams, N. A. An Introduction to Latent Variable Mixture Modeling (Part 2): Longitudinal Latent Class Growth Analysis and Growth Mixture Models. *J. Pediatr. Psychol.* **39**, 188–203 (2014).
34. Jung, T. & Wickrama, K. A. S. An Introduction to Latent Class Growth Analysis and Growth Mixture Modeling. *Soc. Personal. Psychol. Compass* **2**, 302–317 (2008).
35. Gale, C., Ritchie, S. J., Starr, J. M. & Deary, I. J. Physical frailty and decline in general and specific cognitive abilities: The Lothian Birth Cohort 1936. *J. Epidemiol. Community Health* **74**, 108–113 (2019).
36. Filippini, N. *et al.* Study protocol: the Whitehall II imaging sub-study. *BMC Psychiatry* **14**, 159 (2014).
37. Stride, C. *Latent Growth Modelling using Mplus.* (Falcon Publishing: Sheffield, 2016).

© 2020 Anatürk M et al. *JAMA Network Open.*
